# Expanding Current Knowledge on the Chemical Composition and Antioxidant Activity of the Genus *Lactarius*

**DOI:** 10.3390/molecules191220650

**Published:** 2014-12-10

**Authors:** Vanessa Vieira, Lillian Barros, Anabela Martins, Isabel C. F. R. Ferreira

**Affiliations:** 1 Centro de Investigação de Montanha (CIMO), ESA, Instituto Politécnico de Bragança, Campus de Santa Apolónia, Apartado 1172, 5301-855 Bragança, Portugal; E-Mail: vieira.aa.vanessa@gmail.com; 2 School of Agriculture, Polytechnic Institute of Bragança, Campus de Santa Apolónia, Ap. 1172, 5301-855 Bragança, Portugal; E-Mail: amartins@ipb.pt

**Keywords:** *Lactarius citriolens*, *Lactarius turpis*, wild mushrooms, chemical composition, antioxidant activity

## Abstract

Despite the presence of toxic compounds in inedible mushrooms, the question whether the chemical nutrients and non-nutrients compositions in edible and inedible *Lactarius* species are similar remains unanswered. To answer this question, *Lactarius citriolens* Pouzar and *Lactarius turpis* (Weinm.) Fr., two inedible species, were studied in order to obtain information about their chemical composition and bioactivity. Free sugars, fatty acids, tocopherols, organic and phenolic acids were analysed by chromatographic techniques coupled to different detectors. *L. citriolens* and *L. turpis* methanolic extracts were tested regarding antioxidant potential (reducing power, radical scavenging activity and lipid peroxidation inhibition). The composition of macronutrients varied among the two species, but the profiles were similar between them and among other *Lactarius* species; *L. citriolens* gave the highest energy contribution, saturated fatty acids and organic acids, while the *L. turpis* sample was richer in free sugars, mono- and polyunsaturated fatty acids, tocopherols and phenolic compounds. *L. turpis* methanolic extract showed the highest antioxidant activity. The absence of hepatoxicity of the methanolic extracts was confirmed in porcine liver primary cells (*in vitro* conditions). The present study provided new information about wild *L. citriolens* and *L. turpis*, comparing their chemical composition and antioxidant properties with other *Lactarius* species, and expanding the knowledge about this genus.

## 1. Introduction

Wild mushrooms have become more important in our diet for their nutritional [1], organoleptic [2] and medicinal [3] characteristics. The scientific community has studied several mushroom species in order to develop new therapies [3,4]. In fact, they contain a large diversity of compounds with a wide range of nutritional and health benefits such as stimulation of the immune system, providing an anti-cancer function as well as controlling blood lipids in humans [1,3]. The nutraceuticals present in mushrooms have been related with their antioxidant activity [4].

*Lactarius*, popularly known as “milk cap”, is one of the larger genera of ectomycorrhizal *Basidiomycota*, with about 400 species recognized worldwide. Members of the genus are reported in association with numerous trees and shrubs [5]. One particular character of the genus *Lactarius* is that all species exude a typical milky fluid when their basidiocarps are broken. This milky fluid or latex may taste mild or acrid and may be white or colored or may have a changing color depending on the species, providing important taxonomical information [6]. They are considered medicinal and nutritionally important and promising mushrooms [3,4].

*Lactarius citriolens* Pouzar and *Lactarius turpis* (Weinm.) Fr. are inedible species that occur in Bragança, Northeastern of Portugal, and there is not much information about them. Concerning *L. turpis*, there are some studies reporting lead, cadmium and mercury contents [7], antifungal activity against *Penicillium expansum* [8], identification of necatorone (an alkaloid pigment) [9], tolerance to toxic metal minerals [10], and determination of isotope activity (e.g., radiocesium) from contaminated areas [11]. However, nothing has been reported on its chemical composition and bioactivity. Regarding *L. citriolens*, as far as we know, only phylogenetic studies are available in the literature [12].

Our research group has published several works that intend to contribute to inventorying and documenting the chemical and antioxidant properties of wild mushrooms from Northeastern Portugal (including different *Lactarius* species) [13,14,15]. The present work aims to expand the knowledge on the *Lactarius* genus by presenting a detailed chemical characterization of *L. citriolens* and *L. turpis*, including evaluation of nutrients (e.g*.* macronutrients, free sugars, fatty acids and tocopherols), non-nutrients (e.g. phenolic compounds and organic acids) and antioxidant activity of their methanolic extracts (e.g*.* reducing power, radical-scavenging activity and inhibition of lipid peroxidation). The confirmation of non-toxicity of the extracts was performed in a primary cell culture of porcine liver cells.

## 2. Results and Discussion

### 2.1. Chemical Composition of the Fruiting Bodies 

The macronutrients composition of *L. citriolens* and *L. turpis* fruiting bodies is presented in Table 1. *L. turpis* showed the highest level of proteins, ash and carbohydrates, however, the energetic contribution of *L. citriolens* was superior, due to its higher fat content*.* It was possible to find some reports about other species of the same genus, namely *L. deliciosus* [15,16,17,18,19,20,21,22,23], *L. hatsudake* [19], *L. piperatus* [16], *L. quieticolor* [24], *L. salmonicolor* [13,21], *L. sanguifluus* [23,25], *L. semisanguifluus* [23] and *L. volemus* [19]. All of these species also presented carbohydrates and proteins as major macronutrients, and some of them similar energetic contributions (317–389 kcal/100 g dry weight) [13,15]. 

**Table 1:** Macronutrients, free sugars, fatty acids and tocopherols of the fruiting bodies expressed in dry weight basis (mean ± SD).

Parameter	*Lactarius citriolens*	*Lactarius turpis*	*t*-Student Test *p*-value
Fat (g/100 g)	5.37 ± 0.30	2.06 ± 0.27	<0.001
Proteins (g/100 g)	10.89 ± 0.33	13.06 ± 0.29	<0.001
Ash (g/100 g)	6.99 ± 0.23	7.21 ± 0.12	0.109
Carbohydrates (g/100 g)	76.76 ± 0.35	77.68 ± 0.35	0.033
Energy (kcal/100 g)	398.89 ± 1.74	381.47 ± 1.29	<0.001
Mannitol (g/100 g)	8.31 ± 0.30	19.21 ± 0.45	<0.001
Trehalose (g/100 g)	0.45 ± 0.01	0.33 ± 0.03	<0.001
Total sugars (g/100 g)	8.76 ± 0.29	19.54 ± 0.47	<0.001
C16:0	5.35 ± 0.01	8.02 ± 0.09	<0.001
C18:0	40.58 ± 0.41	12.60 ± 0.83	<0.001
C18:1n9	25.00 ± 0.78	26.29 ± 0.98	0.065
C18:2n6	22.46 ± 0.03	48.55 ± 0.14	<0.001
SFA (relative percentage)	51.85 ± 0.70	23.73 ± 1.00	<0.001
MUFA (relative percentage)	25.42 ± 0.78	27.18 ± 0.98	0.026
PUFA (relative percentage)	22.74 ± 0.08	49.09 ± 0.01	<0.001
α-tocopherol (µg/100 g)	20.43 ± 1.27	45.84 ± 5.61	<0.001
β-tocopherol (µg/100 g)	70.65 ± 7.45	14.79 ± 1.54	<0.001
γ-tocopherol (µg/100 g)	4.69 ± 0.70	72.32 ± 7.44	<0.001
δ-tocopherol (µg/100 g)	5.28 ± 0.42	nd	-
Total tocopherols (µg/100 g)	101.05 ± 7.30	132.94 ± 11.50	<0.001

nd‒not detected. Main fatty acids: C16:0 (palmitic acid), C18:0 (stearic acid), C18:1n9 (oleic acid) and C18:2n6 (linoleic acid); 20 more fatty acids were identified in trace amounts. SFA‒saturated fatty acids; MUFA‒monounsaturated fatty acids; PUFA‒polyunsaturated fatty acids.

Observing the macronutrient profile of the genus it can be concluded that the two studied species have similar characteristics to the edible species. However, since there are reports of the presence of necatorin, a highly mutagenic compound, in *L. turpis*, [26], and the possibility of poisoning which manifests by stomach and intestinal troubles [27], *L. turpis* is nowadays considered non-edible [28]. Regarding *L. citriolens*, although this species is rarely found, it should be consumed with caution given the lack of information [28].

**Figure 1:**
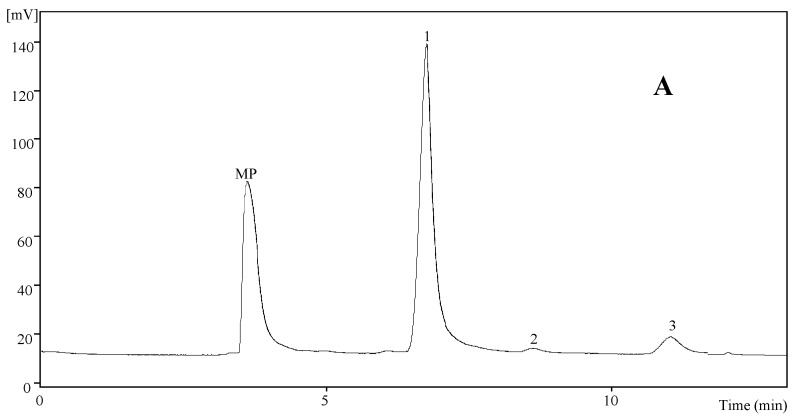

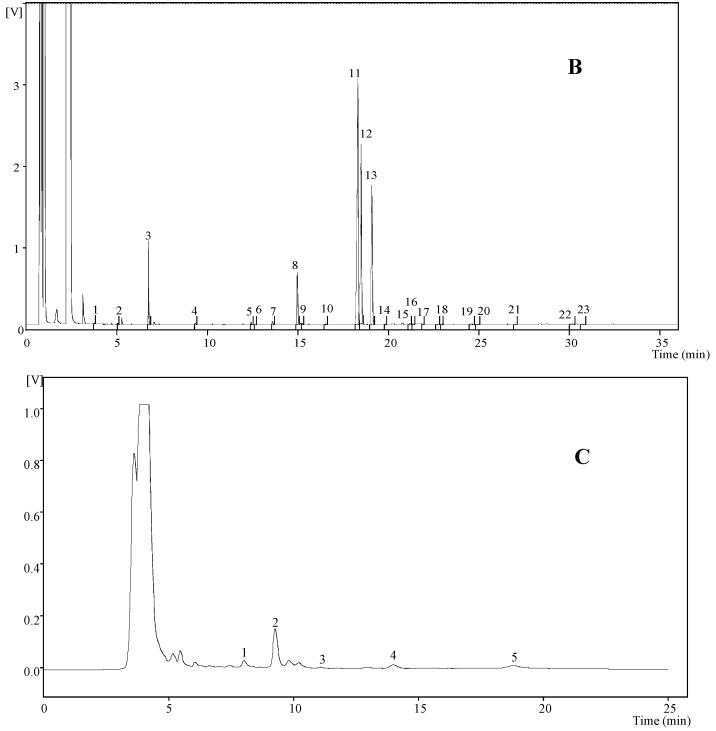
Individual profile in sugars (**A**) of *L. turpis*: 1-mannitol, 2-trehalose, 3-raffinose (IS); fatty acids (**B**) of *L. citriolens*: 1-caproic acid (C6:0); 2-caprylic acid (C8:0); 3-capric acid (C10:0); 4-lauric acid (C12:0); 5-myristic acid (C14:0); 6-myristoleic acid (C14:1); 7-pentadecanoic acid (C15:0); 8-palmitic acid (C16:0); 9-palmitoleic acid (C16:1); 10-heptadecanoic acid (C17:0); 11-stearic acid (C18:0); 12-oleic acid (C18:1n9c); 13-linoleic acid (C18:2n6c); 14-α-linolenic acid (C18:3n3c); 15-arachidic acid (C20:0); 16-eicosenoic acid (C20:1c); 17-cis-11,14-eicosadienoic acid (C20:2c); 18-cis-11,14,17-eicosatrienoic acid and heneicosanoic acid (C20:3n3 + C21:0); 19-cis-5,8,11,14,17-eicosapentaenoic acid (C20:5n3); 20-behenic acid (C22:0); 21-tricosanoic acid (C23:0); 22-lignoceric acid (C24:0); 23-nervonic acid (C24:1); and tocopherols (**C**) of *L turpis*: 1-α-tocopherol, 2-BHT, 3-β-tocopherol, 4-γ-tocopherol, 5-tocol (IS). MP-mobile phase.

Analyzing the free sugars composition, mannitol and trehalose were detected in both samples (Table 1; Figure 1A). *L. turpis* and *L. citriolens* revealed the highest content in mannitol and trehalose, respectively. *L. turpis* gave the highest content in total free sugars. As far as we know, there are only some Portuguese studies, all of them from our research group, on the free sugars in *Lactarius* spp., namely in *L. bertillonii* [29], *L. deliciosus* [15,16,17,18], *L. hepaticus* [30], *L. piperatus* [16], *L. quietus* [14], *L. salmonicolor* [13] and *L. vellereus* [29]. All the mentioned species presented mannitol and trehalose, with the exception of the report of Fernandes *et al.* [15] who also found fructose, but in this case in lower amounts (0.18 g/100 g dry weight). In fact, mannitol, a sugar alcohol, and trehalose, an oligosaccharide, are the main representatives sugars present in mushrooms [1,16]. Mannitol, has half the calories of sucrose and because of their mannitol contents, mushrooms are useful for diabetic patients [31]. Trehalose is a common sugar component of most immature sporocarps and it may function as a reserve material, which can be metabolised when the sporocarps are maturing [32]. 

The fatty acids quantified in higher amounts in both species were palmitic (C16:0), stearic (C18:0), oleic (C18:1n9) and linoleic (C18:2n6) acids (Table 1; Figure 1B). Concerning saturated fatty acids (SFA), palmitic acid was found in higher percentages in *L. turpis*, while stearic acid was presented in higher percentages in *L. citriolens*. Oleic acid (monounsaturated fatty acid-MUFA) and linoleic acid (polyunsaturated fatty acid-PUFA) were found in higher percentages in *L. turpis*.

The *L. citriolens* sample was richer in SFA, however the *L. turpis* sample presented the highest level of MUFA and PUFA. Like *L. citriolens*, there are other *Lactarius* species with stearic acid (C18:0) as the main fatty acid present, namely *L. aurantiacus* [33], *L. bertillonii* [29], *L. deliciosus* [15,16,34], *L. salmonicolor* [13], *L. piperatus* [16] and *L. vellereus* [29]. However, linoleic acid (C18:2) is the major fatty acid in other *Lactarius* species, as occurred in the herein studied *L. turpis* sample, *L. deliciosus* [23,25], *L. hepaticus* [30], *L. quietus* [14], *L. rufus* [35], *L. salmonicolor* [36], *L. sanguifluus* [23,25], *L. semisanguifluus* [23], *L. thejogalus* [35], *L. volemus* [14]. Only one report mentioned oleic acid as the main fatty acid, and it was in a *L. delicious* sample from Portugal [18]. Stearic acid is proved to have a high antibacterial activity since it presents a strong efficacy against Gram-positive and Gram-negative bacteria [37]. Linoleic acid is known as precursor of 1-octen-3-ol, the alcohol of fungi, which is the principal aromatic compound in most fungi [2].

Concerning tocopherols, both samples presented α-, β- and γ-isoforms (Table 1; Figure 1C). The α- and γ-isoforms were the most abundant in *L. turpis*. On the other hand, β-tocopherol was present in higher amounts in *L. citriolens*. This was also the only sample presenting δ-tocopherol. It was *L. turpis* that revealed the highest content of total tocopherols. To our knowledge, there are only Portuguese studies presenting tocopherols content in *Lactarius* spp. The main isoform was variable, and the total content reported varied between 15 µg/100 g to 316 µg/100 g [29,38]. Besides preventing lipid peroxidation, vitamin E appears to exert effects on other cardiovascular risk factors including reduction of platelet adhesion and aggregation [39].

**Figure 2:**
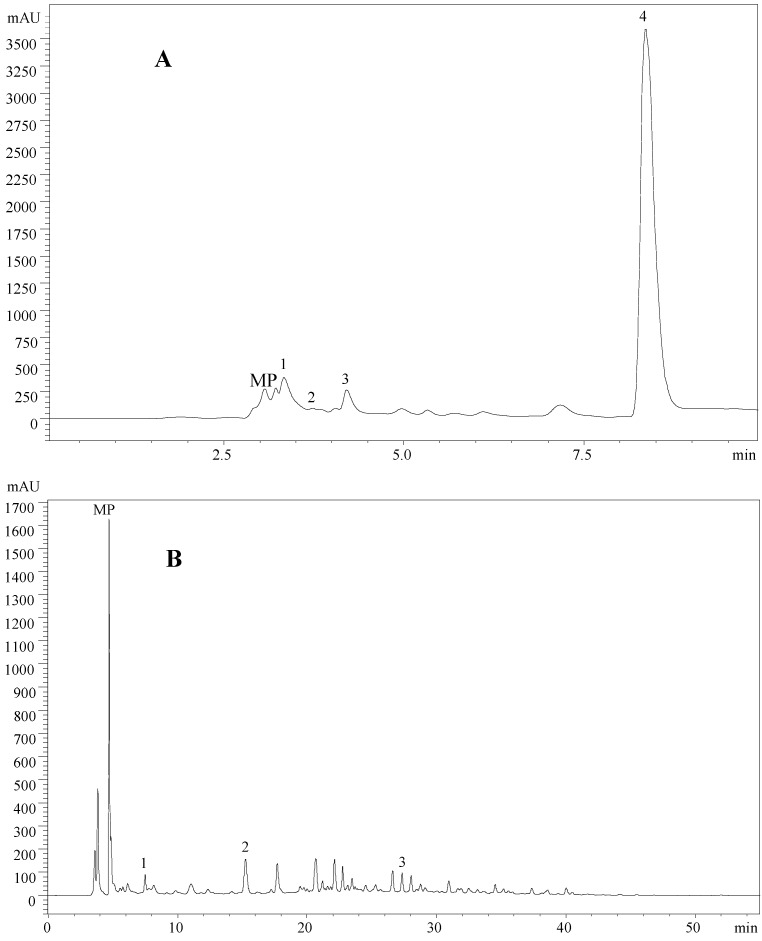
Individual profile in organic acids (**A**) of *L. citriolens*: 1-oxalic acid, 2-quinic acid, 3-malic acid, 4-fumaric acid; and phenolic acids (**B**) of *L. turpis*: 1-gallic acid; 2-*p*-hydroxybenzoic acid; 3-cinnamic acid. MP-mobile phase.

Regarding the organic acids profile, it was possible to identify and quantify four different compounds (Table 2; Figure 2A), namely oxalic, quinic, malic and fumaric acids. The wild mushroom *L. turpis* was the one that revealed the highest concentration of oxalic acid, while *L. citriolens* presented the highest malic and fumaric acids content. Quinic acid was only present in *L. citriolens* and this mushroom also showed the highest content in total organic acids.

**Table 2:** Organic acids and phenolic compounds of the fruiting bodies expressed in dry weight basis (mean ± SD).

Compound	*Lactarius citriolens*	*Lactarius turpis*	*t*-Student test *p*-value
Oxalic acid (g/100 g)	0.06 ± 0.01	0.10 ± 0.01	<0.001
Quinic acid (g/100 g)	0.18 ± 0.06	nd	-
Malic acid (g/100 g)	3.36 ± 0.03	2.96 ± 0.19	0.008
Fumaric acid (g/100 g)	0.50 ± 0.02	0.24 ± 0.00	<0.001
Total organic acids (g/100 g)	4.10 ± 0.06	3.30 ± 0.19	<0.001
Gallic acid (mg/100 g)	nd	0.08 ± 0.00	-
*p*-Hydroxybenzoic acid (mg/100 g)	0.15 ± 0.01	0.12 ± 0.00	0.002
Total phenolic acids (mg/100 g)	0.15 ± 0.01	0.20 ± 0.01	<0.001
Cinnamic acid (mg/100 g)	0.15 ± 0.00	0.12 ± 0.00	<0.001

nd—not detected.

As far as we know, there are only two reports concerning organic acids in *Lactarius* spp. namely, *L. deliciosus* from different locations within Portugal and *L. volemus*, with malic acid as the main organic acid present [40,41].

Concerning phenolic acids, the studied samples revealed the presence of gallic and *p*-hydroxybenzoic acids, and also the related compound cinnamic acid (Table 2; Figure 2B). Gallic acid was only found in *L. turpis*. However, it was *L. citriolens* that presented the highest amounts of *p*-hydroxybenzoic acid and of the related compound cinnamic acid. Analyzing these results we can conclude that the wild *L. turpis* sample was richer in phenolic acids compared to *L. citriolens*. It is difficult to establish a profile for the genus as for what concerns phenolic compounds. In fact, the phenolic compounds recorded in different species are not the same and the main phenolic compounds also vary among different species. For example, *p*-hydroxybenzoic acid was the main phenolic acid in *L. deliciosus* [42], *L. salmonicolor* [43] and *L. volemus* [14]. However, *ο*-coumaric [23], homogentisic [44] and tannic [45] acids were also reported as the main phenolic compounds in* L. deliciosus* and *L. volemus* samples. For *L. volemus*, protocatechuic acid was also reported as the main phenolic acid [46]. The same phenolic acid was in major abundance in *L. vellereus* [29]; catechin was also found in *L. deterrimus* [47] and *L. vellereus* [48]; and *ο*-coumaric was the major phenolic acid in *L. semisanguifluus* [23]. *p*-OH-Phenylacetic acid was the main phenolic acid in *L. sanguifluus* [23], however Puttaraju *et al*. [45] reported tannic acid as the principal compound for the same species. Regarding the related compound cinnamic acid, it was found in *L. aurantiacus* [43], *L. bertillonii* [29] and *L. quietus* [14]. In fact, *Lactarius* spp. is a genus rich in phenolic compounds, which have been reported to display different health benefits.

### 2.2. Antioxidant Activity of the Methanolic Extracts and Confirmation of Non-Toxicity

Analyzing the results of the antioxidant potential (Table 3), *L. turpis* methanolic extract revealed the highest reducing power evaluated through the Folin-Ciocalteu and ferricyanide/Prussian blue assays. The same sample also revealed the highest radical scavenging activity, since it presented the lowest EC_50_ value for DPPH assay. *L. turpis* also showed the highest lipid peroxidation inhibition in the β-carotene/linoleate and TBARS assays. 

**Table 3:** Antioxidant activity of the methanolic extracts (mean ± SD).

Antioxidant Activity	Assay	*Lactarius citriolens*	*Lactarius turpis*	*t*-Student Test *p*-Value
Reducing power	Folin-Ciocalteu (mg GAE/g extract)	13.13 ± 0.17	22.02 ± 0.09	<0.001
	Ferricyanide/Prussian blue (EC_50_; mg/mL)	2.61 ± 0.13	1.53 ± 0.02	<0.001
Radical scavenging activity	DPPH scavenging activity (EC_50_; mg/mL)	15.77 ± 0.27	4.18 ± 0.04	<0.001
Lipid peroxidation inhibition	β-Carotene/linoleate (EC_50_; mg/mL)	6.21± 0.24	4.92 ± 0.27	<0.001
	TBARS (EC_50_; mg/mL)	0.82 ± 0.03	0.57 ± 0.02	<0.001

Concerning the Folin-Ciocalteu assay, higher values mean higher reducing power (GAE-gallic acid equivalents); for the other assays, the results are presented in EC_50_ values, what means that higher values correspond to lower reducing power or antioxidant potential. EC_50_: Extract concentration corresponding to 50% of antioxidant activity or 0.5 of absorbance for the Ferricyanide/Prussian blue assay.

The highest total antioxidant activity in the genus, measured through Folin-Ciocalteu assay, was reported by Orhan and Üstün [49] in a *L. deliciosus* sample (51.27 mg GAE/g). *L. deliciosus* (EC_50_ = 500 µg/mL; [49]) and *L. bertillonii* (EC_50_ = 1.63 mg/mL; [29]) gave the highest reducing power. Regarding radical scavenging activity, the highest capacity was described by Unekwu *et al*. [50] for a *L. deliciosus* sample (EC_50_ = 300 µg/mL). Finally, regarding the lipid peroxidation inhibition, the highest antioxidant activity was obtained in *L. deliciosus* (IC_50_ = 148.0 µg/mL) measured by β-carotene/linoleate assay [34] and *L. bertillonii* (EC_50_ = 1.21 mg/mL) through TBARS assay [29]. Both of our samples present lower EC_50_ values in the TBARS assay being *L. turpis* the species with the highest antioxidant potential of the genus measured by this biochemical assay. Antioxidants can help the endogenous defense system, playing an important role as potential protective agents, reducing oxidative damage by free radicals so, preventing events related to aging and diseases, such as atherosclerosis, diabetes, cancer and cirrhosis [4].

As the methanolic extracts displayed antioxidant activity, it was important to evaluate their cytotoxicity against liver cells, which are considered the best *in vitro* model for studies of human cytotoxicity. Despite of the reported presence of toxic compounds in *L. turpis* fruiting bodies, the prepared extracts did not reveal any toxicity in PLP2 liver primary culture; the positive control ellipticine gave a GI_50_ (sample concentration that inhibited 50% of the net cell growth) = 2.06 ± 0.03 µg/mL. This could indicate that the toxic compounds were not present in these extracts. Nevertheless, it should be highlighted that an *in vitro* assay was performed, and that under *in vivo* conditions the microbiota that inhabit our intestines can readily convert molecules, some of which becoming toxic. 

## 3. Experimental Section 

### 3.1. Sampling of Mushroom Species

*Lactarius citriolens* Pouzar and *Lactarius turpis* (Weinm.) Fr. samples were collected in Bragança (Northeastern Portugal), in November 2012. The authentications were done at the Polytechnic Institute of Bragança. Voucher specimens were deposited at herbarium of School of Agriculture of Polytechnic Institute of Bragança, Portugal. The samples were immediately lyophilised (FreeZone 4.5, Labconco, Kansas City, MO, USA), reduced to a fine dried powder (20 mesh), mixed to obtain a homogeneous sample and stored in a desiccator, protected from light, until further analysis. 

### 3.2. Chemical Composition of L. citriolens and L. turpis Fruiting Bodies 

#### 3.2.1. Macronutrients. 

The samples were analysed for moisture, proteins, fat, carbohydrates and ash using the standard procedures [51]. The crude protein content (N × 4.38) of the samples was estimated by the macro-Kjeldahl method; the crude fat was determined by extracting a known weight of powdered sample with petroleum ether, using a Soxhlet apparatus; the ash content was determined by incineration at 600 ± 15 °C. Total carbohydrates were calculated by difference. Energy was calculated according to the following equation: Energy (kcal) = 4 × (g protein + g carbohydrate) + 9 × (g fat). 

#### 3.2.2. Individual Compounds

Free sugars were determined by a high performance liquid chromatograph (HPLC) system coupled to a refraction index (RI) detector as previously described by the authors [13]. Fatty acids were determined after a transesterification procedure as described previously by the authors [13]. The fatty acids profile was analyzed using a gas chromatographer equipped with a flame ionization detector (GC-FID). Tocopherols were determined following a procedure previously described by the authors [13]. Analysis was performed by HPLC (equipment described above), and a fluorescence detector. Organic acids were determined by ultra-fast liquid chromatography (UFLC) coupled with a photodiode array detector (PDA) as previously described by the authors [41]. Phenolic acids determination was performed using the UFLC mentioned above, as previously described by Barros *et al.* [42]. 

### 3.3. Bioactivity of L. citriolens and L. turpis Methanolic Extracts

#### 3.3.1. Extract Preparation

Each lyophilized sample (1 g) was extracted by stirring with methanol (40 mL) for 1 h and subsequently filtered through Whatman No. 4 paper. The residue was then extracted with methanol (20 mL) for 1 h. The combined methanolic extracts were evaporated at 40 °C (Büchi R-210 rotary evaporator, Flawil, Switzerland) to dryness and re-dissolved in: (a) methanol for antioxidant activity assays (20 mg/mL) and (b) distillated water for the toxicity assay in porcine liver primary cells (8 mg/mL).

#### 3.3.2. Antioxidant Activity Assays

The antioxidant activity of the methanolic extracts was evaluated by DPPH radical-scavenging activity, reducing power (Folin-Ciocalteu and ferricyanide/Prussian blue assays), inhibition of β-carotene bleaching in the presence of linoleic acid radicals and inhibition of lipid peroxidation using TBARS in brain homogenates. Trolox was used as positive control [13]. 

#### 3.3.3. Toxicity for Porcine Liver Cells

The assay was performed with a cell culture prepared from a freshly harvested porcine liver (PLP2), and by applying sulphorhodamine assay. A complete procedure was previously described by the authors [52]. Ellipticine was used as positive control.

### 3.4. Statistical Analysis

Three samples were used for each preparation and all the assays were carried out in triplicate. The results are expressed as mean values and standard deviation (SD). The results were analyzed using a t-student test and this treatment was carried out using the SPSS v. 22.0 program. 

## 4. Conclusions 

Overall, *L. citriolens* revealed the highest energetic contribution, saturated fatty acids content and organic acids concentration, while *L. turpis* was richer in free sugars, mono- and polyunsaturated fatty acids, tocopherols and phenolic compounds. *L. turpis* methanolic extract showed the highest antioxidant activity in all the *in vitro* assays. The studied extracts did not show toxicity in porcine liver primary cells. The edible species of the *Lactarius* genus are considered healthy foods due to their low content in calories and fat, but richness in proteins and carbohydrates. The macronutrients profiles of *L. citriolens* and *L. turpis* are similar to the ones reported for those edible species, despite the presence of some toxic compounds that make them not recommended for consumption. Nevertheless, they contain several interesting molecules with bioactive potential, namely antioxidant activity, which can be isolated to be used in drugs or nutraceuticals. This study provides new data concerning chemical characterization and bioactivity of *L. citriolens* and *L. turpis*. 
